# A collaborative approach to training ward nurses in acute care skills in resource limited settings: the nursing intensive care skills training (nicts) project

**DOI:** 10.1186/2197-425X-3-S1-A445

**Published:** 2015-10-01

**Authors:** A Beane, T Stephens, AP De Silva, M Adikaram, S De Alwis, P Athapattu, C Sigera, L Peiris, S Siriwardana, KSA Jayasinghe, A Dondorp, R Haniffa

**Affiliations:** Adult Critical Care Department, Barts Health NHS Trust, London, United Kingdom; Critical Care Research Team, Barts Health NHS Trust, London, United Kingdom; Queen Mary University of London, William Harvey Institute, London, United Kingdom; National Intensive Care Surveillance, Colombo, Sri Lanka; Ministry of Health, Office of Deputy Director General (Education, Training and Research), Colombo, Sri Lanka; Nursing Council of Sri Lanka, Colombo, Sri Lanka; Department of Clinical Medicine, Faculty of Medicine, University of Colombo, Colombo, Sri Lanka; Mahidol Oxford Tropical Medicine Research Unit (MORU), Bangkok, Thailand

## Introduction

Early recognition and prevention of deterioration of ward patients can improve patient outcomes and reduce critical care admissions [1]. In low and middle income countries (LMICs), with often minimal access to critical care therapies, the benefit may be even greater. However training to assist ward nurses develop acute care skills remains limited in such settings. As part of the NICST portfolio of acute care training, the Sri Lankan nursing faculty sought assistance to deliver a 2 day course for ward nurses [2].

## Objectives

To design a clinically relevant short course for ward nurses in a LMIC to be delivered by local nursing tutors and facilitators.

To assess whether such a clinically focused programme would increase ward nurses' knowledge and skills in identifying and managing deteriorating patients.

## Methods

A multi-modal 2 day acute care course for ward nurses was co-designed and delivered by specialist overseas trainers in partnership with national tutors. The courses were sponsored by the Ministry of Health, Sri Lanka. Based upon the NICST model of collaborative course design, local faculty were up skilled in delivery and content through a pre course Train the Trainer programme [3].

Candidates were invited to undertake on-line pre course e-learning. Core clinical guidelines were delivered using mini lectures. Facilitator-led skills stations and structured scenarios were used to develop clinical skills.

Short term knowledge acquisition was tested by a pre and post course Multi-Choice Questionnaire (MCQ). Newly acquired skills and their application was assessed through a post course Objective Clinical Skills Assessment (OSCA) station.

## Results

122 ward nurses were trained over 6 courses in 2 locations. Post MCQ scores were significantly higher for each course compared to pre MCQ (Wilcoxon sign rank test P < 0.0001).

Over 71% passed the OSCA (pass mark of 60). Feedback reveals high candidate satisfaction.

## Conclusions

Our short course results demonstrate an increase in relevant knowledge and clinical skills of the participants. Our NICST model demonstrates the feasibility of a local nursing faculty in a LMIC co-designing and effectively delivering a setting adapted acute care training programme integrated into the local nurse training system.

**Table 1:** Ward course pre and post course results.

Time	Venue	Number of faculty members trained	Number of trainees	Pre Course MCQ mean (SD)	Post course MCQ mean (SD)	P value
2014 June	Colombo	21	31	66.3 (14.8)	80.3 (11.2)	0.0007
2014 September	Colombo	20	14	53.2 (9.3)	67.4 (9.9)	0.0016
2014 October	Moneragala	16	27	51.0 (14.4)	73.3 (11.8)	0.0001
2014 November	Colombo	13	13	52.0 (7.0)	65.3 (8.6)	0.0074
2015 March	Moneragala	11	20	46.8 (10.6)	65.8 (7.9)	0.0001
2015 March	Colombo	18	17	48.9 (14.6)	67.6 (12.7)	0.0144

**Table 2:** Selected course candidate feedback.

Evaluation	Strongly agree' and 'agree' combined					
	June (n = 27)	Sept (n = 8)	Oct (n = 23)	Nov (n = 13)	March PBCN (n = 11)	March M'ragala (n = 20)
I acquired new knowledge and skills	27 (100%)	7 (87.5%)	22 (95.7%)	13 (100%)	11 (100%)	20 (100%)
The sessions were supported with adequate demonstration of intensive care skills	25 (96.1%)	7 (87.5%)	22 (100%)	13 (100%)	11 (100%)	20 (100%)
The information in lectures was conveyed clearly	23 (85.2%)	8 (100%)	21 (95.5%)	13 (100%)	11 (100%)	19 (95%)
I got adequate opportunity for hands-on experience	24 (92.3%)	7 (87.5%)	20 (90.9%)	12 (92.3%)	11 (100%)	15 (88.2%)
I found the content relevant to my practice	24 (100%)	7 (87.5%)	21 (100%)	13 (100%)	11 (100%)	20 (100%)
